# Enhanced Mechanical Stability of Gold Nanotips through Carbon Nanocone Encapsulation

**DOI:** 10.1038/srep10408

**Published:** 2015-06-17

**Authors:** Abraham G. Cano-Marquez, Wesller G. Schmidt, Jenaina Ribeiro-Soares, Luiz Gustavo Cançado, Wagner N. Rodrigues, Adelina P. Santos, Clascidia A. Furtado, Pedro A.S. Autreto, Ricardo Paupitz, Douglas S. Galvão, Ado Jorio

**Affiliations:** 1 Departamento de Física, ICEx, Universidade Federal de Minas Gerais, Belo Horizonte, MG, 31270-901, Brazil; 2 Materials Research Institute, N-261 Millennium Science Complex, Penn State University, University Park, PA 16802, USA; 3 Centro de Microscopia, Universidade Federal de Minas Gerais, Belo Horizonte, MG, 30123-970, Brazil; 4 Centro de Desenvolvimento da Tecnologia Nuclear, Belo Horizonte, MG, 31270-010, Brazil; 5 Instituto de Física ‘Gleb Wataghin’, Universidade Estadual de Campinas, Campinas, SP, 13083-970, Brazil; 6 Departamento de Física, IGCE, Universidade Estadual Paulista - UNESP, Rio Claro, SP, 13506-900, Brazil

## Abstract

Gold is a noble metal that, in comparison with silver and copper, has the advantage of corrosion resistance. Despite its high conductivity, chemical stability and biocompatibility, gold exhibits high plasticity, which limits its applications in some nanodevices. Here, we report an experimental and theoretical study on how to attain enhanced mechanical stability of gold nanotips. The gold tips were fabricated by chemical etching and further encapsulated with carbon nanocones via nanomanipulation. Atomic force microscopy experiments were carried out to test their mechanical stability. Molecular dynamics simulations show that the encapsulated nanocone changes the strain release mechanisms at the nanoscale by blocking gold atomic sliding, redistributing the strain along the whole nanostructure. The carbon nanocones are conducting and can induce magnetism, thus opening new avenues on the exploitation of transport, mechanical and magnetic properties of gold covered by sp^2^ carbon at the nanoscale.

Ductility and malleability are two aspects of plasticity. A solid is ductile if it easily deforms under tensile stress, and malleable if it easily deforms under compressive stress. The focus of this paper is on gold,^1^ which is both ductile and malleable^2^,^3^,^4^,^5^. By observing a gold nanojunction in the process of breaking by mechanical pulling inside an electron microscope, researchers were able to show the gold atoms moving around almost freely^6^. One can thus ask how to mechanically stabilize gold at the atomic scale, to be used in nanoelectronics, nanoelectromechanics or nanoplasmonics. For example, metal nanotips are used in scanning tunneling microscopies (STM)^7^,^8^, but gold is definitely not a preferable option, precisely due to its mechanical fragility. However, the use of gold may be important, like in photonics, as nanoantennas^9^,^10^. In nano-optics, the geometrical shape of the tip rules the conductivity, while surface roughness, cone angle, curvature radius and level of crystallinity define the efficiency of the tip-enhanced Raman scattering process^11^,^12^,^13^,^14^,^15^. However, the mechanical instability makes the fabrication and use of gold nanotips an irreproducible and highly inefficient procedure. Finding alternatives for combining the advantages of gold (chemical stability, biocompatibility, electrical and optical conductivity) with a robust mechanics is a technological challenge.

A single-walled carbon nanocone (SWCNC) is topologically a graphene sheet rolled up into a cone^16^. They exhibit unique structural, mechanical, thermal, chemical, and electronic properties, typical of sp^2^ related carbons, but they can be even stiffer than carbon nanotubes^16^. Theoretically, there are five different types of SWCNTs, with different apex angles, which vary according to the different number of pentagons (also called disclinations), determined during nucleation processes^17^. These carbon nanocones can have micrometer-size bases and tip radii ranging from less than one nanometer up to several tens of nanometers. They can be composed of one (SWCNC) or several layers, the later named multi-walled carbon nanocones (MWCNCs). Sharp carbon nanocones were first reported in 1994, and proposed as mechanically stable tips for scanning probe microscopy^18^. They were subsequently synthesized with different structures^19^. Annealed cones, in contrast to their non-annealed counterparts, present large crystallite sizes, less structural disorder, and better electrochemical performance^20^. Furthermore, pentagons and other polygons at the tip apex induce magnetism due to the appearance of unpaired electrons^17^ and sharp resonant peaks in the local density of states (LDOS)^21^. The enhanced LDOS in the vicinity of the pentagonal rings can be exploited in applications, such as field emission sources^21^ and scanning probes^22^.

In Fig. 1 we show how to build a new type of nanotip that combines the properties of the gold and carbon nanostructures discussed above. An electrochemically-etched gold nanotip was encapsulated with a MWCNC with the aid of a dual beam microscope and nanofabrication facility (see Fig. 1). The gold nanotip was glued with cyanoacryllic adhesive into a steel needle, which was then placed in a nanomanipulator and inserted into the microscope chamber. Next, an isolated MWCNC was located on top of a Si substrate (Fig. 1A). The gold nanotip was then approached and inserted into the MWCNC, as shown in Fig. 1B,C, and then soldered with platinum, as shown in Fig.1D. During tip assembly, both electron and gallium ion beams were used to align the gold nanotip and the nanocone. The carbon nanocone encapsulated gold tip (from now on named Au@CNC) can thus be easily handled for further manipulations.

To test the hybrid system mechanically, we mounted the Au@CNC tip on a tuning fork, to be used as a probe for atomic force microscopy (AFM) (see Fig.2A). Several topography scans could be performed without losing resolution, even using relatively high tip-sample feedback control values that are prohibitive for bare gold tip scans. Figure 2B,C show topography images obtained by scanning single-walled carbon nanotubes deposited on glass with the Au@CNC tip, while Fig. 2D shows the height profile along the blue trace in Fig. 2C. The Au@CNC tip was damaged only under extreme compression conditions, by turning off the AFM feedback control and pressing the Au@CNC tip against the substrate (see details in the Supplemental Information).

The nature of the enhanced mechanical stability of the Au@CNC tip as compared with the bare gold tip is revealed by fully atomistic reactive molecular dynamics simulations, as summarized in Fig. 3. The bare gold tip deforms quite easily under compression, as indicated by the insets on top of upper part of Fig. 3 (I-IV). During this process, the bare gold tip overall stress (red curve) is highly concentrated near the contact area between the tip and the substrate, as indicated by the stress color scale within the atoms in the respective insets. Taking into account that during these deformations several atomic rearrangements take place, and that atomic planes slide against each other, we can see that the system is not in the elastic regime and the deformations occur irreversibly (see also video01(a,b) in the Supplementary Materials).

On the other hand, in the case of Au@CNC, the black curve and the corresponding insets at the bottom of Fig. 3 (I-V) show that the mechanical behavior is completely different. There are 5 phases for the compression process, schematically represented in the snapshots at the bottom insets to Fig. 3: (I) encapsulation process. The Au@CNC tip is structurally stable, held together by van der Waals interactions; (II-III) Au@CNC interacting directly with the substrate and also showing the elastic deformation at the CNC apex. At the same time that the CNC is deformed against the substrate, the Au tip is tightly pushed against the nanocone; (III-IV) a non-elastic deformation with partial folding of carbon layers at the CNC apex takes place. Consequently, gold deforms inside the nanocone causing a compact filling of this carbon structure. The experienced stress on the gold tip is lower and well distributed over the whole structures. Just before IV the strain in the gold atoms increases fast since the atomic movements are blocked by the nanocone. The white colored atoms at the top of the tip (see snapshot IV at the bottom of Fig.3) indicates the stress is distributed over the entire gold tip structure; (V) after the compression is released (when the Au@CNC tip is retracted), the cone structure almost recovers its original atomic tip apex configuration. Most importantly, the gold tip does not show permanent deformation. The whole process can be visualized in the video02(a,b) of the Supplementary Materials.

The attained enhanced structural stability of the Au@CNC tip is a consequence of the CNC blocking the gold atomic sliding, while at the same time preserving its elastic behavior and redistributing the stress over a larger volume. Of course, if the Au@CNC is pushed beyond a certain pressure limit, it can be fractured with the formation of linear chains of carbon atoms, which appear in *in situ* Raman spectroscopy measurements, indicating tip-induced field enhancement^23^, and in simulations (see Supplementary Materials, including video03). Interestingly, the fractured structures seem to be oxidation resistant even in the presence of high content oxygen atmosphere (see video04 in Supplementary Materials). The fabrication of this new hybrid Au@CNC tip thus solves the mechanical problem that has limited the use of gold as nanotips, achieving its ultimate enhanced structural stability. This opens new and interesting perspectives for a broader use of these enhanced performance gold tips in many nanotechnological applications.

## Methods Summary

### Fabrication of Au@CNC nanotips

Annealed carbon cones were purchased from N-TEC (Norway). Purification of the nanocone samples was performed combining thermal treatments^24^, suspension and centrifugation procedures. Electrochemical etching was used in a simple electrochemical cell, which consisted of a ring of platinum wire as counter-electrode, and a high purity, annealed gold wire (0.113 mm d., 99.998%) as working electrode. At the dual beam microscope (FEI Quanta 3D), the parameters for image acquisition were: electron beam, E_acc_ = 5 kV, spot = 4.5; ion beam, E_acc_ = 30 kV, I = 0.10 nA. Before soldering the cone to the tip, it is recommended to open the Pt source with the nanotip not yet inserted, since this action may make the Au tip to move with a jerk, with subsequent loss – in many cases – of the nanocone by hitting. In some cases, sharpening of the tip with gallium ion beam had to be done for the gold tip to be able to properly fit into the cone.

### Experimental characterization

AFM experiments were performed with a home-made piezo-controlled scan head and a scanning probe microscopy controller. The gold tip is mounted onto a tuning fork, which is placed on the piezo-controlled scan head. The sample to be analyzed is placed over a nanometer-precise positioning stage (Nano-H50, MadCityLabs). The tested Au@CNC tip lasted up to one month of intense use, much more than conventional non-modified gold tips, without losing image resolution and keeping greater stability. Unsoldered Au@CNC tips exhibited unstable scanning performance. *In situ* Raman spectroscopy (561.4 nm laser) was performed on a home-made system, which consists of an inverted optical microscope equipped with a high-aperture optical objective (Nikon, 60X, 1.4 NA), to characterize the carbon nanocone.

### Computational Methods

Fully atomistic molecular dynamics (MD) simulations using the reactive force field ReaxFF^25^ were carried out using the large-scale atomic/molecular massively parallel simulator (LAMMPS) code^26^. We have always used a NVT ensemble controlled by a Nose-Hoover thermostat^27^. ReaxFF, which is parameterized using DFT calculations, employs a bond length/bond order relationship for use in MD calculations, allowing the simulation of chemical reactions. All bond orders are calculated for each simulation step, and charge effects are taken into account using EEM approach^28^,^29^. In the case of gold containing systems, the DFT calculations for the parameterization were based on PBE exchange-correlation potential calculations^30^. In order to describe the evolution and distribution of stress in the considered structures, for each time step of the simulations we calculated the virial stress tensor and the *von Mises stress*^31^.

## Additional Information

**How to cite this article**: Cano-Marquez, A. G. *et al.* Enhanced Mechanical Stability of Gold Nanotips through Carbon Nanocone Encapsulation. *Sci. Rep.* **5**, 10408; doi: 10.1038/srep10408 (2015).

## Supplementary Material

Supplementary Information

Supplementary Video 1

Supplementary Video 2

Supplementary Video 3

Supplementary Video 4

Supplementary Video 5

Supplementary Video 6

## Figures and Tables

**Figure 1:**
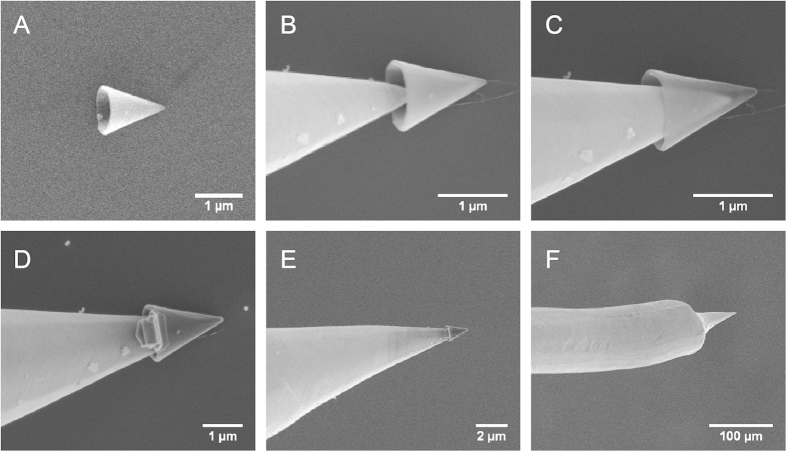
Encapsulation of a gold nanotip with a carbon nanocone. **** (**A**) One isolated MWCNC on Si substrate; (**B**-**D**) Approaching and soldering MWCNC on a gold tip; (**E**, **F**) Zoom-out views showing the Au nanotip on a larger scale; In (**F**) the nanocone is no longer seen.

**Figure 2:**
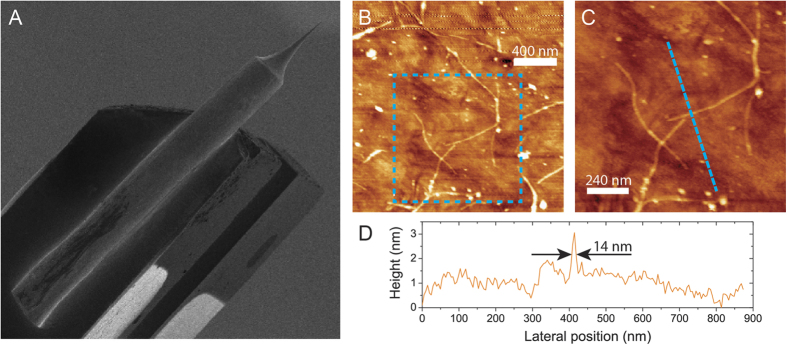
Atomic force microscopy (AFM) obtained with a gold nanotip encapsulated with a carbon nanocone (Au@CNC) . (**A**) SEM image of an Au@CNC tip glued into a tuning fork for AFM measurements; (**B**,**C**) Raw AFM topography images of single wall carbon nanotubes on a glass cover slip; (**D**) Height profile along the blue dashed line in (**C**), showing lateral resolution better than 14 nm.

**Figure 3:**
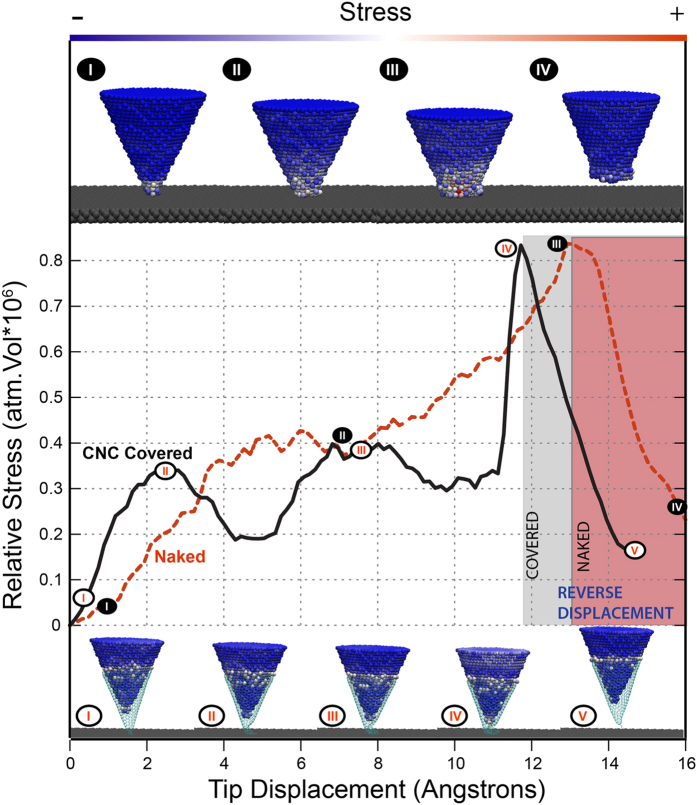
Molecular dynamics simulations of pushing a bare and an encapsulated (Au@CNC) gold tips against a solid substrate. **** The red (bare tip) and black (encapsulated tip) curves show the stress-strain (experienced by the gold tip) as a function of simulation time. Top and bottom insets are representative snapshots of the moments indicated in the black and red curves, respectively. The shaded area refers to the moment where the tip starts to be retracted.
