# Supplementary material for: Enhanced Mechanical Stability of Gold Nanotips through Carbon Nanocone Encapsulation
Source: Sci Rep. 2015 Jun 17;5:10408. doi: 10.1038/srep10408 (PMC4470435; doi:10.1038/srep10408)
Supplement: Supplementary Information [file srep10408-s1.pdf]

Supplementary Information: Enhanced Mechanical Stability of Gold Nanotips through Carbon Nanocone Encapsulation

Abraham G. Cano-Marquez[1,2], Wesller G. Schmidt[3], Jenaina Ribeiro-Soares[1], Luiz Gustavo Cançado[1], Wagner N. Rodrigues[1], Adelina P. Santos[4], Clascidia A. Furtado[4], Pedro A.S. Autreto[5], Ricardo Paupitz[6], Douglas S. Galvão[5*], Ado Jorio[1*]

[1]*Departamento de Física, ICEx, Universidade Federal de Minas Gerais, Belo Horizonte, MG, 30123-970, Brazil;*
[2] *Materials Research Institute, N-261 Millennium Science Complex, Penn State University, University Park, PA 16802, USA;*
[3] *Centro de Microscopia, Universidade Federal de Minas Gerais, Belo Horizonte, MG, 30123-970, Brazil;*
[4] *Centro de Desenvolvimento da Tecnologia Nuclear, Belo Horizonte, MG, 31270-010, Brazil;*
[5]*Instituto de Física 'Gleb Wataghin', Universidade Estadual de Campinas, Campinas, SP, 13083-970, Brazil.*
[6]*Departamento de Física, IGCE, Universidade Estadual Paulista - UNESP, Rio Claro, SP, 13506-900, Brazil.*

**Corresponding authors:* adojorio@fisica.ufmg.br and galvao@ifi.unicamp.br

Purification of carbon microdisks and nanocones

Annealed carbon cones (2500 °C to 2700 °C) were provided by N-TEC (Norway). These cones were synthesized by pyrolysis of heavy oil at 2000 °C (International Patent No. WO 98/42621). A typical sample contains about 20% of annealed cones, from which there are five different types according to their apex angle (19.2°, 38.9°, 60°, 83.6°, and 112.9°). Purification of N-TEC pristine sample was performed combining thermal treatments, suspension and centrifugation procedures. Thermal treatments were carried on the basis of the work by Jiménez-Soto and coworkers[1]. An alternative method was sonication of the NTEC material in a sodium dodecylbenzene aqueous solution for over 3 days, followed by fraction separation by a 90-min centrifugation at 1000 RPM and 10 °C (Sorvall). The material at the bottom and low fractions contained the highest content of carbon microdisks and cones with just a few graphitic particles present.

Fabrication of AFM gold nanotips by electrochemical etching

Electrochemical etching was used in a simple electrochemical cell, which consisted of a ring of platinum wire (Aldrich, 2 mm wire d., 99.95%) as counter-electrode, and a high purity, annealed (800 °C, 1 – 2 h, argon) gold wire (Alfa Aesar, 0.113 mm d., 99.998%) as working electrode. Since control over the etching current and time is crucial[1], we used a control system. An electric potential of +4.7 V was applied by means of a power source (Minipa 4201A) through a digital acquisition interface (DAQ, National Instruments), in an intermittently way during 20 ms periods and controlled by a virtual feedback circuit designed with LabView software. The feedback circuit helps to decrease the electric current that is being applied to the gold wire. The circuit opens when the gold wire breaks, thus automatically stopping the etching process.

Nanomanipulation and SEM imaging

A Dual Beam Microscope (FEI Quanta 3D) was used for this work. A high acceleration voltage and low current (pico- to nanoampere range) were set at the Ga^+ beam, since high currents destroy or severely damage the carbon nanocone. The parameters for image acquisition were: electron beam, E_{acc} = 5 kV, spot = 4.5; ion beam, E_{acc} = 30 kV, I = 0.10 nA. Depending on the area to be soldered, platinum deposition depth may vary between 200 nm and 2000 nm. Before soldering the cone to the tip, it is recommended to open the Pt source with the nanotip not yet inserted, since this action may make the Au tip to move with a jerk, with subsequent loss – in many cases – of the nanocone by hitting. In some cases, sharpening of the tip with gallium ion beam had to be done for the gold tip to be able to properly fit into the cone.

Some examples of failure

Figure S1 shows some examples of failure on the process of making Ac@CNC nanotips. The reasons are impurities attached to the tips and inadequate Au tip-carbon nanocone geometry matching.

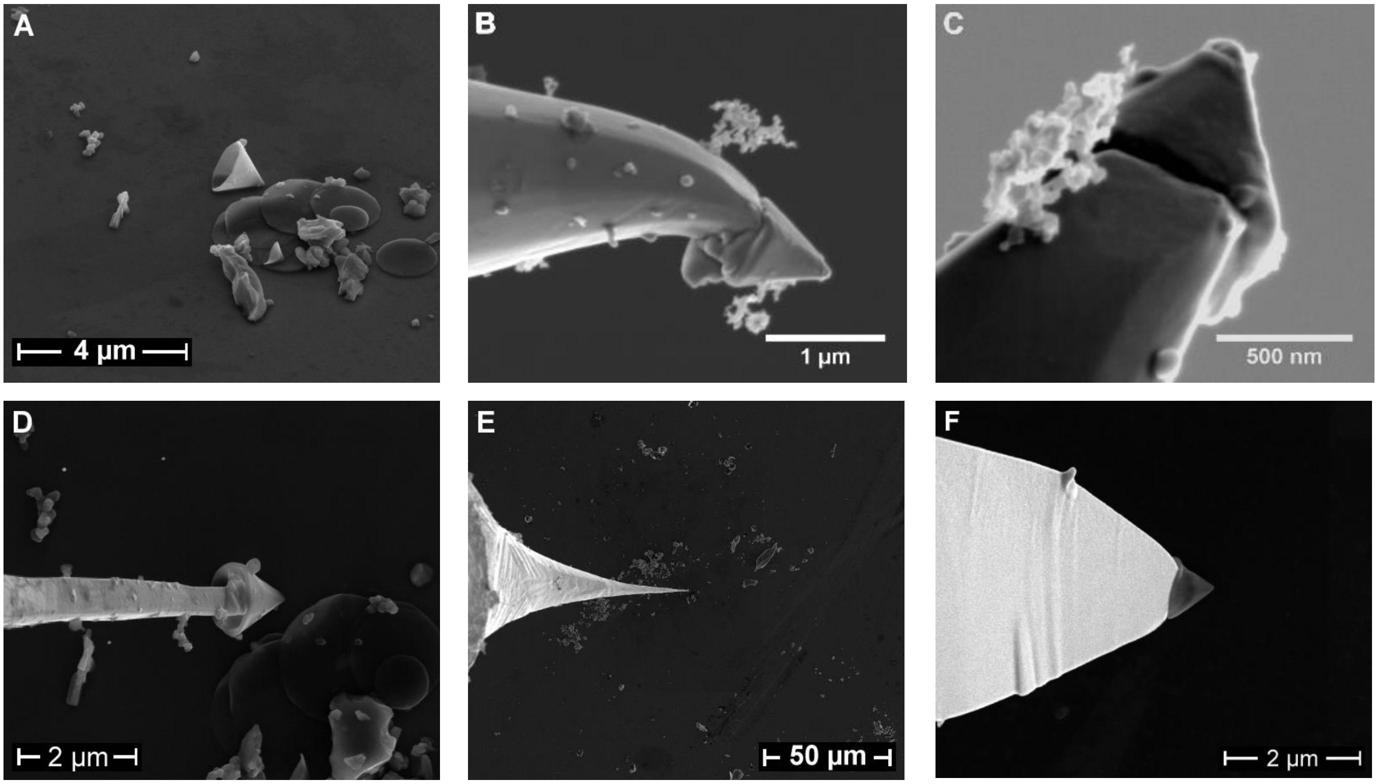

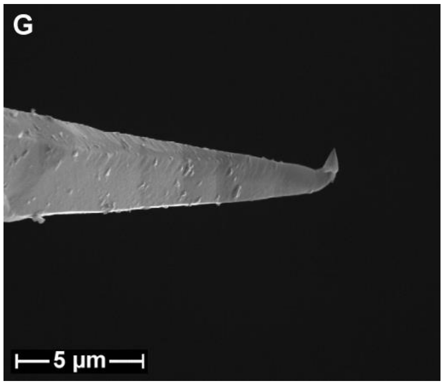

Figure S1. *In A a carbon nanocone deposited on a Si substrate is shown, near carbon impurities. B and C show two Au@CNC tips with carbon impurities that destroy the mechanical stability. D and E show an Au tip too long and sharp to exhibit a good match with the carbon nanocone. F shows an Au tip too "fat" to fit into the carbon nanocone. G shows a distorted soldering that was inadequate to our setup, but that could be a good geometry for different systems.*

Atomic Force microscopy (AFM)

AFM experiments were performed with a home-made scanning probe microscopy (SPM) system. The gold tip is mounted onto a tuning fork, which is placed on the piezo-controlled scan head. The sample to be analyzed is placed over a nanometer-precise positioning stage (Nano-H50, MadCityLabs). The x-y movement and scanned area of this stage is controlled by the user via software (XPM pro 2.0.1.5). The software commands an electronic signal generator (RHK Systems), which sends a varying voltage to an electronic controller (Nano Drive 2, MadCityLabs). In this way we can vary the scanned area (up to 40 μm^2), exposure time at each point, scan speed, and image resolution. The tested Au@CNC tips lasted up to one month of frequent use, even using relatively close tip-sample scanning sets, and without losing image resolution. Unsoldered Au/cone tips exhibited unstable scanning performance.

Confocal Raman imaging

Raman spectroscopy was performed on the home-made system, which consists of an inverted optical microscope equipped with a high-aperture optical objective (Nikon, 60X, 1.4 NA), an xy-scan stage, a Raman spectrometer (Andor) coupled to a CCD (iDUS) and a single-photon avalanche photodiode (APD from Perkin Elmer). The AFM setup described above can be placed on top of the microscope, thus allowing *in situ* Raman spectroscopy and AFM imaging. Confocal Raman imaging acquired with the APD is a point-by-point accurate raster image of the carbon structure being analyzed. The sample to be analyzed is placed over a nanometer-precise positioning stage (Nano-H50, MadCityLabs). The x-y movement and scanned area of this stage is controlled by the user via software (XPM pro 2.0.1.5). Confocal images were acquired by placing filters in front of the APD; to acquire an image of the G band in the sample, we placed a 620 nm filter (561.4 nm laser). Analysis on the characteristic Raman shifts of a multiwalled carbon nanocone and a multiwalled carbon microdisk were done upon varying the power of a 561.4 nm laser. Raman spectra were acquired under the following conditions: laser line: 561.4 nm (yellow-green), slit: 50 µm, grating: 600 l/mm, acquisition time = 30 s, number of accumulations = 6. The spectrometer was previously calibrated with a Neon lamp using 640.22 nm as central wavelength.

For more detailed structural information, we performed *in situ* Raman spectroscopy experiments, focusing on the MWCNT signal, as shown in Fig. S2. A few works have already reported the Raman features of carbon nanocones, where the typical first- and second-order Raman signals from sp^2 nanocarbons were identified, corresponding to the first-order features D, G and D' bands (at 1340, 1580 and 1620 cm^{-1} for a 632.8 nm laser), and at 2660 and 3240 cm^{-1} as second-order G' and 2D' features[2-5]. Figure S2 A shows a typical Raman spectrum of one isolated MWCNC sitting on the Si substrate. It corresponds to the cone in Fig.S2 E, which shows a confocal Raman imaging where the intensity of the G band is displayed. The intensity ratio of the G and G' bands remains practically constant by measuring at different locations of this MWCNC, the relative intensity and the shape of the G' band being characteristic of a multilayered, turbostratic graphitic system (compare with Fig.S2 D for highly ordered pyrolytic graphite). Raman spectra acquired with low power (6 µW) showed no significant changes with respect to the position of the bands along the carbon nanocone. On the other hand, spectra acquired with higher power (95 µW) showed a notable shift on the Raman frequencies, in the order of 10 – 20 cm^{-1}, due to non-homogeneous laser heating of the MWCNC. The highest redshifts are located at the cone edge and apex, indicating these

defective regions cannot dissipate heat as fast as the central part of the cones. In all cases we can see a very small contribution of the D band, which can be more clearly noticed at the cone edge, in agreement with a defective (more reactive[6]) structure.

Figure S2 B shows the Raman spectrum of one isolated multiwalled carbon nanocone mounted on the Au@CNC nanotip. The spectrum is very different from the ones from the unmounted MWCNC (Fig.S2 A), because the way the system is mounted, we are probing the nanocone tip apex. The system is settled in a back-scattering configuration, with the laser traveling in the tip axis direction, i.e. directly towards the tip apex. The electric field is perpendicular to the propagation direction, i.e. polarized in the tip apex plane. Broad first-order Raman features D and G bands around 1339 and 1587 cm^{-1} are seen, as well as and a wide band at 1488 cm^{-1}, probably related to the pentagons at the cone apex. The second-order features are washed out. For comparison, we show the Raman spectra of the bulk MWCNC sample before purification. The sample is composed by highly graphitic disks (70% w), carbon nanocones (20%), impurities and amorphous carbon (10%). The spectrum in Fig. S2 C clearly reflects a superposition of the spectral information of these materials. Interesting is the more well defined peak at 1461cm^{-1}, assigned here as the cone apex response.

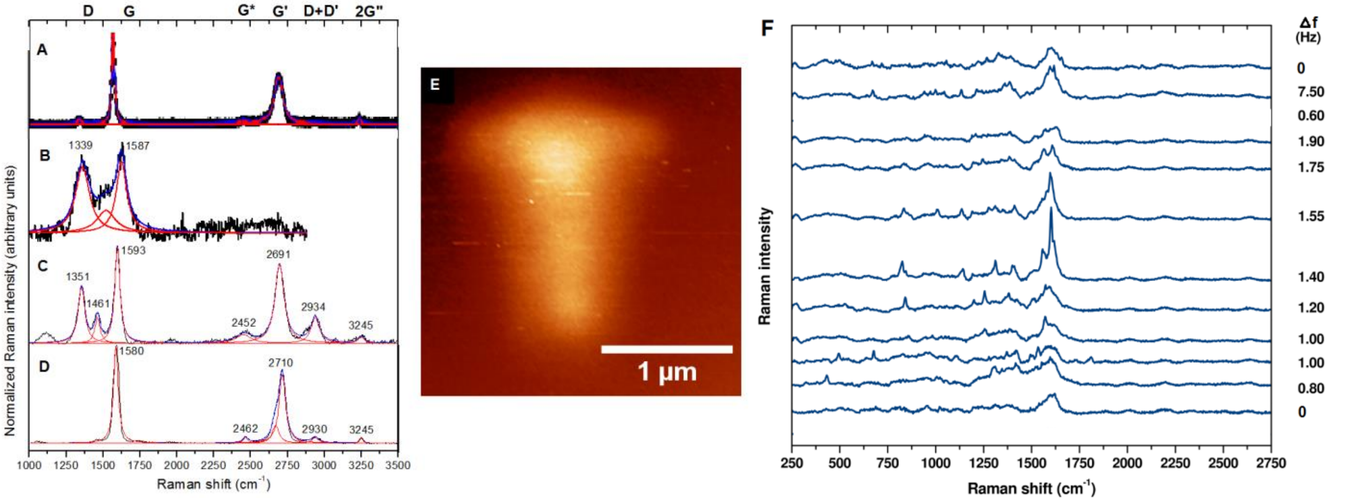

Figure S2. *Raman spectrum from an isolated multiwalled carbon nanocones sitting on the silicion substrate (A), and placed on a gold tip – the Au@CNC (B); (C) Raman spectrum from the N-TEC sample consisting of 70 wt% carbon nanodisks, 20% wt multiwalled carbon nanocones and 10% wt impurities; (D) Raman spectrum from graphite for comparison. (E) Confocal Raman imaging of the isolated MWCNC on the silicon substrate shown in (A). Laser excitation at λ = 561.4 nm. (F) In situ Raman spectroscopy measurements of an Au@CNC tip being smashed against a glass substrate. The Raman spectra were taken during the increase and decrease of the cone-substrate interaction. The frequency values at the right side correspond to the change in frequency of the tuning fork upon approaching the tip to the substrate, thus representing the degree of interaction of the tip with the substrate.*

To test the stability of the system under extreme compression conditions, we turned off the AFM feedback control and pressed the Au@CNC tip against the substrate. *In situ* Raman measurements were performed during the procedure, and some results are shown in Fig S2 F. Upon pressing the cone against the glass

substrate we can see a dynamical and unstable apparition of several sharp Raman features in the range from 300 to 1600 cm^{-1}. Similar results have been broadly observed in the TERS community and ascribed as due to amorphous carbon in the sample[7]. Our experiment indicates the formation of short-carbon chain species upon interaction of the carbon nanocone with the substrate[8-11]. The small bands in the range 690 – 1400 cm^{-1} might originate from –C-H stretching or –C-C- out of plane vibrations stretching vibrations usually found in unsaturated carbons[9,10]. We can also speculate that these bands could be due to the generation of polyynes[12] or cumulenic species[13], which could be stabilized by a transition metal, this is, the gold already present in the tip[13]. The broad band at 500 and the peak at 1310 cm^{-1} (D band) are related to –C-C- stretching in sp^3 carbon found in amorphous carbon[9]. The formation of nitrogen, oxygen and hydrogen compounds upon reaction of carbon atoms with air lie within the 2000 to 3000 cm^{-1} region, and are therefore excluded from our analysis.

Computational Methods

The dynamical aspects of gold nanotips under mechanical deformations were studied using fully atomistic molecular dynamics (MD) techniques using the reactive force field ReaxFF[14], as implemented in the large-scale atomic/molecular massively parallel simulator (LAMMPS) code[15], always considering a NVT ensemble controlled by a Nose-Hoover thermostat[16].

ReaxFF, which is parameterized using DFT calculations, employs a bond length/bond order relationship for use in MD calculations, allowing the simulation of chemical reactions. In some aspects, this potential is similar to standard non-reactive force fields, since the system energy is divided into partial energy contributions, which include bonded and non-bonded terms[14]:

$$E_{system} = E_{bond} + E_{over} + E_{under} + E_{val} + E_{pen} + E_{tors} + E_{conj} + E_{vdWaals} + E_{coulomb},$$

where E_{bond} represents the bond energy, E_{over} is an over-coordination term, E_{under} a under-coordination one, E_{val} the valence energy, E_{tors} torsion energy, $E_{vdWaals}$ van der Waals energy and $E_{Coulomb}$ the Coulomb energy. There are also an energy penalty for handling atoms with two double bonds, E_{pen} and a conjugated bond energies term E_{conj}.

In order to obtain the reactivity during the simulations, all bond orders are calculated for each simulation step, and charge effects are taken into account using EEM approach[17,18]. In the case of gold containing systems, the DFT calculations for the parameterization were based on PBE exchange-correlation potential calculations. This set of parameters was conceived to allow the simulation of large scale systems containg gold, hydrogen, sulfur and carbon atoms[19]. The average deviation between the ReaxFF predictions and the PBE results for these systems is relatively small, for instance, for AuS it was found to be 0.06 eV/Atom and for H_3CS-Au-SCH_3 0.38 eV/Atom[19].

Stress Calculations

In order to describe the evolution and distribution of stress in the considered structures, for each time step of the simulations we calculated the virial stress tensor[20], defined as

$$\sigma_{ij} = \frac{\sum_{k}^{N} m_k v_{k_i} v_{kj}}{V} + \frac{\sum_{k}^{N} r_k . f_k}{V}$$

where *N* is the number of atoms, *V* is the volume, *m* the mass of the atom, *v* is the velocity, *r* is the position and *f* the force acting on the atom. We also calculated a quantity, which is related to the distortion state of the system, known as *von Mises stress*[21] and defined as:

$$\sigma_{vm} = \sqrt{\frac{(\sigma_{11} - \sigma_{22})^2 + (\sigma_{22} - \sigma_{33})^2 + (\sigma_{11} - \sigma_{33})^2 + 6(\sigma_{12} + \sigma_{23} + \sigma_{31})^2}{V}}$$

This quantity provides very helpful information on fracturing processes because, by calculating it for each timestep, it is possible to visualize the time evolution and localization of stress on the structure, as well as to identify regions where fractures are more likely to occur.

Supplementary Videos

Video01a – Bare gold nanotip structural deformation
Molecular Dynamics Simulation of bare gold nanotip structural deformation when pressed against a rigid surface.
Video01b – Bare gold nanotip structural deformation and atomic stress
Molecular Dynamics Simulation of bare gold nanotip structural deformation when pressed against a rigid surface. The atomic color code delivers the dynamical local stress felt by the atoms during the process.

Video02a – Carbon nanocone-gold nanotip structural deformation
Molecular Dynamics Simulation of carbon nanocone-gold nanotip structural deformation when pressed against a rigid surface.
Video02b – Carbon nanocone-gold nanotip structural deformation and atomic stress
Molecular Dynamics Simulation of carbon nanocone-gold nanotip structural deformation when pressed against a rigid surface. The atomic color code delivers the dynamical local stress felt by the atoms during the process.

Video03 – Rigid gold nanotip destroying carbon nanocones
Molecular Dynamics Simulation of rigid gold nanotip destroying carbon nanocones.

Video04 – Carbon nanocone-gold nanotip structural deformation in air
Molecular Dynamics Simulation of carbon nanocone-gold nanotip structural deformation when pressed against a rigid surface, in the presence of oxygen molecules.

REFERENCES

1. Jiménez-Soto, J. M., Cárdenas, S. & Valcárcel, M. Evaluation of carbon nanocones/disks as sorbent material for solid-phase extraction. *J. Chromatogr. A* **1216,** 5626-5633 (2009).

2. Jaszczak, J. A., Robinson, G. W., Dimovski, S. & Gogotsi, Y. Naturally occurring graphite cones. *Carbon* **41,** 2085-2092 (2003).

3. Shang, N., Milne, W. I. & Jiang, X. Tubular graphite cones with single-crystal nanotips and their antioxygenic properties. *J. Am. Chem. Soc.* **129,** 8907-8911 (2007).

4. Tan, P.H., Zhang, J., Wang, X.C., Zhang, G.Y. & Wang, E.G. Raman scattering from an individual tubular graphite cone. *Carbon* **45,** 1105-1136 (2007).

5. Zhang, G., Jiang, X. & Wang, E. Tubular graphite cones. *Science* **300**, 472-474 (2003).

6. Park, S., Srivastava, D. & Cho, K. Generalized chemical reactivity of curved surfaces: carbon nanotubes. *Nano Lett.* **3**, 1273-1277 (2003).

7. Chaigneau, M., Picardi, G. & Ossikovski, R. Tip enhanced Raman spectroscopy evidence for amorphous carbon contamination on gold surfaces. *Surface Science* **604,** 701–705 (2010).

8. Kip, B. J. et al. Considerations for Raman spectroscopic determination of polyene length distribution in degraded poly(vinyl chloride). *Macromolecules* **25,** 4290-4296 (1992).

9. Lenz, J. A., Perottoni, C. A., Balzaretti, N. M. & da Jornada, J. A. H. Processing of amorphous carbon films by ultrafast temperature treatment in a confined geometry. *J. Appl. Phys.* **89,** 8284-8290 (2001).

10. Parker, W. L., Siedle, A. R. & Hexter, R. M. Raman spectroscopy of unsaturated hydrocarbons on supported rhodium and palladium. *Langmuir* **4,** 999-1006 (1988).

11. Schaffer, H. E., Chance, R. R., Silbey, R. J., Knoll, K. & Schrock, R. R. Conjugation length dependence of Raman scattering in a series of linear polyenes: Implications for polyacetylene. *J. Chem. Phys.* **94,** 4161-4170 (1991).

12. Casari, C. S. et al. Low-frequency modes in the Raman spectrum of sp-sp2 nanostructured carbon. *Phys. Rev. B* **77,** 1954441-1954447 (2008).

13. Gu, X., Kaiser, R. I. & Mebel, A. M. Chemistry of energetically activated cumulenes— from allene (H2CCCH2) to hexapentaene (H2CCCCCCH2). *ChemPhysChem* **9,** 350-369 (2008).

14. Van Duin, A. C. T., Dasgupta, S., Lorant, F. & Goddard III, W. A. ReaxFF: A Reactive force field for hydrocarbons. *J. Phys. Chem. A* **105,** 9396-9409 (2001).

15. Plimpton, S. Fast Parallel algorithms for short-range molecular dynamics. *J. Comp. Phys.* **117,** 1-19 (1995).

16. Martyna, G. J., Tuckerman, M. E., Tobias, D. J. & Klein, M. L. Explicit reversible integrators for extended systems dynamics. *Mol. Phys.* **87,** 1117-1157 (1996).

17. Janssens, G. O. A. et al. Comparison of cluster and infinite crystal calculations on zeolites with the electronegativity equalization method (EEM). *J. Phys. Chem.* **99,** 3251-3258 (1995).

18. Mortier, W. J., Ghosh, S. K. & Shankar, S. Electronegativity equalization method for the calculation of atomic charges in molecules. *J. Am. Chem. Soc.* **108,** 4315-4320 (1986).

19. Järvi, T. T., Van Duin, A. C. T., Nordlund, K. & Goddard III, W. A. Development of Interatomic ReaxFF potentials for Au-S-C-H Systems. *J. Phys. Chem. A* **115,** 10315-10322 (2011).

20. Subramaniyan, A. K. & Sun, C. T. Continuum interpretation of the virial stress in molecular simulations. *Int. J. Solids Struct.* **45,** 4340-4346 (2008).

21. Garcia, A. G. & Buehler, M. J. Bioinspired nanoporous silicon provides great toughness at great deformability. *Comput. Mater. Sci.* **48,** 303-309 (2010).
